# Pulmonary arterial wall disease in COPD and interstitial lung diseases candidates for lung transplantation

**DOI:** 10.1186/s12931-017-0568-z

**Published:** 2017-05-06

**Authors:** Enric Domingo, Juan C. Grignola, Rio Aguilar, Manuel López Messeguer, Antonio Roman

**Affiliations:** 1 Area del Cor, Hospital Universitari Vall d’Hebron, Barcelona, Spain; 2 Physiology Department, School of Medicine, Universitat Autonoma, Barcelona, Spain; 3 Pathophysiology Department, School of Medicine, Hospital de Clínicas, Universidad de la República, Avda Italia 2870, PC 11600 Montevideo, Uruguay; 4 Cardiology Department, Hospital Universitario de la Princesa, Universidad Autónoma de Madrid, Madrid, Spain; 5 Department of Neumology, Hospital Universitari Vall d’Hebron, Barcelona, Spain; 6 Ciberes, Instituto de Salud Carlos III, Madrid, Spain

**Keywords:** Pulmonary hypertension, Lung transplantation, Pulmonary arterial wall, Chronic obstructive pulmonary disease, Interstitial lung disease

## Abstract

**Background:**

Pulmonary hypertension (PH) associated with lung disease has the worst prognosis of all types of PH. Pulmonary arterial vasculopathy is an early event in the natural history of chronic obstructive pulmonary disease (COPD) and interstitial lung disease (ILD). The present study characterized the alterations in the structure and function of the pulmonary arterial (PA) wall of COPD and ILD candidates for lung transplantation (LTx).

**Methods:**

A cohort of 73 patients, 63 pre-LTx (30 COPD, 33 ILD), and ten controls underwent simultaneous right heart catheterisation and intravascular ultrasound (IVUS). Total pulmonary resistance (TPR), capacitance (Cp), and the TPR-Cp relationship were assessed. PA stiffness and the relative area of wall thickness were estimated as pulse PA pressure/IVUS pulsatility and as [(external sectional area-intimal area)/external sectional area] × 100, respectively.

**Results:**

Twenty-seven percent of patients had pulmonary arterial wedge pressure > 15 mmHg and were not analyzed. PA stiffness and the area of wall thickness were increased in comparison with controls, even in patients without PH (*p* < 0.05). ILD patients showed a significant higher PA stiffness, and lower Cp beyond mean PA pressure (mPAP) and lower area of wall thickness than COPD patients (*p* < 0.05). TPR-Cp relationship was shifted downward left for ILD patients.

**Conclusions:**

Significant increase of PA stiffness and area of wall thickness were present even in patients without PH and can make the diagnosis of pulmonary vasculopathy at a preclinical stage in PH-lung disease candidates for LTx. ILD patients showed the worst PA stiffness and Cp with respect to COPD.

## Background

Pulmonary hypertension (PH) is a well-recognized complication of chronic obstructive pulmonary disease (COPD) and interstitial lung disease (ILD) that impacts on the functional capacity, the risk of hospitalization, and survival [1]. PH associated with lung disease has the worst prognosis of all types of PH [2]. The prevalence of PH in both chronic lung diseases depends on the severity of the disease and the definition and methodologies used in the studies [3, 4]. Most of the available data regarding PH-lung disease comes from patients undergoing evaluation for lung transplantation (LTx). In a recent cohort of 362 patients with severe COPD who were evaluated for LTx and underwent right heart catheterization, the prevalence of pre-capillary PH was 23% [5, 6]. PH may complicate the course of many forms of ILD [7, 8]. The prevalence of PH for idiopathic pulmonary fibrosis (IPF), the most frequent of ILD disease, is between 10 to 80% [9–11].

The relation between the severity and extent of pulmonary vascular disease and the degree of hemodynamic abnormalities seen in PH-lung disease is poorly understood [1]. Recently, the analysis of pulmonary arterial lesions in explanted lungs of COPD patients demonstrated that histological pulmonary vascular changes are prevalent, including lesions Grade 3 (Heath & Edwards) even in patients without PH [12]. The analysis of the United Network for Organ Sharing database showed that an increase in the mean pulmonary arterial pressure (mPAP) from the time of wait listing to the time of LTx was associated with poorer survival in patients with COPD and ILD after LTx even in the lung allocation scoring era [6, 13]. Therefore, the early diagnosis of pulmonary vascular disease and PH in COPD and ILD candidates for LTx could have an impact on survival.

We hypothesized that pulmonary vascular disease is present even with normal resting mPAP and would be related to the hemodynamic and parenchymal disease severity (obstructive/restrictive impairment) in COPD and ILD candidates for LTx. The first aim of the present study is to assess and to compare the extent of functional (pulmonary artery stiffness, PAS) and anatomical (area of wall thickness, AWT) pulmonary vascular disease in COPD and ILD candidates for LTx according to the hemodynamic severity. The second aim was to correlate the pulmonary arterial vasculopathy with the severity of airway and parenchymal lung impairment in COPD and ILD candidates for LTx, respectively.

## Methods

### Study design

A cohort of 63 consecutive adult subjects candidates for LTx for COPD (*n* = 30) and ILD (*n* = 33) between January 2011 and December 2012 was included. All patients were studied as potential candidates for lung transplantation according to the International Society for Heart and Lung Transplantation guidelines [14]. Evaluation included clinical and laboratory examination, chest X-ray imaging, pulmonary function tests (forced expiratory volume in one second, FEV1; the forced vital capacity, FVC; diffusion capacity for carbon monoxide, DLCO), electrocardiography, 6-min walk test (6MWT), Doppler echocardiography, and high-resolution computed tomography imaging. All patients underwent a routine right heart catheterization and simultaneous medium-sized elastic pulmonary arteries intravascular ultrasound (IVUS) in the supine position and breathing room air.

The patients were compared with ten control subjects referred for cardiac catheterization due to clinically suspected PH, and in whom PH and other respiratory and cardiovascular diseases were discarded. PH was defined as mPAP greater or equal than 25 mmHg.

The study complied with the Declaration of Helsinki and the study protocol was approved by the Institutional Ethics Committee of the Hospital Universitari Vall d’Hebron (Barcelona). Written informed consent was obtained from all patients.

### Hemodynamic and IVUS studies

A 7 F Swan-Ganz catheter was inserted into a brachial vein, and a 5 F end-hole catheter was inserted into the right radial artery to monitor systemic arterial pressure. Both catheters were connected to pressure transducers. The zero reference level for recording was at mid-thoracic level [15]. An electrocardiogram and heart rate were recorded continuously, and cardiac output (CO) was assessed using the indirect Fick method. Resting oxygen uptake was calculated by the formula of Dehmer, based on body surface area (BSA) (125 mL/min/m^2^ × BSA) [16]. All pressures, including right atrial pressure (RAP), mPAP and pulmonary arterial wedge pressure (PAWP), were taken in the supine position and at end-expiration [17]. We calculated pulmonary vascular resistance (PVR) and total pulmonary resistance (TPR) as (mPAP-PAWP)/CO and mPAP/CO, respectively. Total pulmonary arterial capacitance (Cp) was estimated by the stroke volume/pulse PAP ratio [18]. We discarded the patients with PAWP ≥ 15 mmHg. We estimated pulmonary arterial RC time as the product of resistance and capacitance (PVR × Cp). We examined the relationship between TPR and Cp to analyze the consequences of pulmonary vascular disease to the dynamic afterload [19]. To compare the TPR-Cp relationship for each group, the TPR, and Cp data were logarithmically transformed and plotted on a linear axis.

IVUS examination was performed with an Opticross imaging catheter 40 MHz, 3.1 F (Boston Scientific Corporation, USA) with an axial resolution of 38 μm. A minimum of three representative images were obtained from segmental pulmonary arteries (elastic pulmonary artery ~ 2–3 mm of diameter) [20, 21] and stored in digital form. Both diastolic and systolic cross-sectional areas of the studied segment were analyzed off-line by two observers unaware of clinical and hemodynamic findings. We estimated IVUS pulsatility (IVUSp) as (systolic-diastolic lumen area)/diastolic lumen area × 100. PAS was assessed by the elastic modulus (EM) or pressure/elastic strain index (pulse PAP/IVUSp), an expression of the intrinsic pulmonary arterial wall viscoelastic properties [22]. Intra- and inter-observer validation of IVUS measurements in our laboratory have been previously published [20, 21]. Because of the strong dependency of strain on underlying pressures, we compared Cp and EM of both groups according to mPAP [22]. Finally, we calculated the relative AWT as (external sectional area-intimal area)/external cross-sectional area × 100 [23].

### Statistical analysis

Continuous variables are expressed as mean ± SEM. Categorical variables were summarized by frequency. Independent sample t-tests were used to compare differences between the control, COPD and ILD patients. Chi-squared was used for comparing proportions of patients. Intergroup variation was analyzed using one-way ANOVA.

The association between vascular remodeling (EM and AWT), hemodynamic impairment and lung functional tests were explored using linear regression analysis (Pearson coefficient). A two-sided *P* value < 0.05 was regarded as significant. Data analysis were carried out using SPSS 17.0 for Windows 7 software.

## Results

Twenty-seven percent of patients had PAWP > 15 mmHg (*n* = 17) and were excluded. Pre-capillary PH was presented in 44% of COPD (8/18) and 46% of ILD (13/28). COPD and ILD patients presented an unimodal distribution with a mean of 26 ± 2 and 27 ± 2 mmHg, respectively, indicating that PH was mainly mild to moderate when present.

Baseline demographics, clinical data, and pulmonary function test were shown in Table 1. Both, COPD and ILD patients were older than the control group (*p* < 0.05). The proportion of women was 5% in COPD, 36% in ILD and 60% in Controls (*p* < 0.05). However, body mass index was not different between the groups. The aetiologies of ILD correspond to Idiopathic Pulmonary Fibrosis (61%); Idiopathic non-Specific Interstitial Pneumonitis (10%); Connective Tissue Disease-related ILD (10%) and others (19%) [24].

**Table 1:** Demographic, anthropometric, clinical, and pulmonary function data in control subjects and patients with COPD and ILD

	COPD (*n* = 18)	ILD (*n* = 28)	Control (*n* = 10)
Age, years	58 ± 1*	58 ± 2*	51 ± 2
Sex, M/F	17/1*	18/10*§	4/6
BMI, kg/m^2^	25.0 ± 1.4	26.3 ± 0.8	23.6 ± 1.2
FC II/III/IV, %	0/78/22	14/79/7	
6MWT, meters	300 ± 22	315 ± 15	
FVC, L	1.8 ± 0.2	1.9 ± 0.14	
FVC, % predicted	44 ± 3	50 ± 3	
FEV1, L	0.77 ± 0.08	1.56 ± 0.13§	
FEV1, % predicted	24 ± 2	58 ± 3§	
FEV1/FVC	0.42 ± 0.03	0.82 ± 0.03§	
DLCO, % predicted	33 ± 5	27 ± 2§	
FVC%/DLCO%	1.5 ± 1.0	2.6 ± 0.3§	
PaO_2_, mm Hg	64 ± 2.3	62 ± 2.4	
PaCO_2_, mm Hg	50 ± 2.3	40 ± 1.4§	

Data are mean ± SEM

*Abbreviations*: *BMI* body mass index, *6MWT* 6-min walking test, *DLCO* diffusion capacity of the lung for carbon monoxide, *FC* functional class, *FEV1* forced expiratory volume in one second, *FVC* forced vital capacity, *PaO*_*2*_ *and PaCO*_*2*_ partial arterial pressure of oxygen and dioxide of carbon, respectively

*p < 0.05 vs. Control; §*p* < 0.05 vs. COPD

Both, functional class and 6MWT showed no differences between COPD and ILD patients. All patients showed relative hypoxemia with normo (ILD) or hypercapnia (COPD). Both groups showed a severely reduced FVC, and DLCO had higher impairment in ILD than in COPD patients (*p* < 0.05), with out of proportion to the reduction in lung volume (FVC%/DLCO%). PH patients walked less than non-PH patients (COPD: 288 ± 28 versus 307 ± 34 m; and ILD: 280 ± 16 versus 345 ± 22 m), although only in ILD group reached to statistical significance. PH patients presented significant lower partial pressure of arterial oxygen (PaO_2_) than non-PH patients (COPD: 59 ± 2 versus 65 ± 2 mmHg; and ILD: 57 ± 2 versus 66 ± 2 mmHg). Both, FEV1 in COPD patients, and FVC in ILD patients did not show significant differences between PH and non-PH patients.

### Pulmonary hemodynamics and pulmonary arterial wall disease in COPD and ILD

ILD patients showed significantly higher pulse PAP and lower Cp than in COPD patients, in spite of similar mPAP, which is in relation with a lower RC time (Table 2).

**Table 2:** Hemodynamic and IVUS data in control subjects and patients with COPD and ILD with or without pulmonary hypertension

	Chronic Obstructive Pulmonary Disease			Interstitial Lung Disease			Control
	All (18)	Non PH (10)	PH (8)	All (28)	Non PH (15)	PH (13)	(10)
mPAP, mm Hg	26 ± 2*	21 ± 1*	33 ± 3*#	27 ± 2*	20 ± 1*	32 ± 3*	15 ± 2
pPAP, mm Hg	16 ± 2*	14 ± 1	18 ± 3*	23 ± 2*§	17 ± 0.9*	27 ± 4*#§	11 ± 3
CI, L/min/m^2^	2.5 ± 0.2	2.3 ± 0.2*	2.5 ± 0.2	2.5 ± 0.1	2.5 ± 0.1	2.5 ± 0.1	2.6 ± 0.1
HR, bpm	76 ± 3	72 ± 4	82 ± 4.4	81 ± 2.6	80 ± 4	83 ± 3	73 ± 1.3
SV, mL	57 ± 2	54 ± 3	56 ± 4	60 ± 2	61 ± 3	59 ± 3	64 ± 2
RAP, mm Hg	8.7 ± 0.7*	7.4 ± 0.7*	10.8 ± 1.2*#	5.4 ± 0.6§	5.2 ± 0.8	6.1 ± 0.4§	5.0 ± 1.2
PAWP, mm Hg	10.9 ± 0.7	10.0 ± 1.0	11.6 ± 0.7*	8.0 ± 0.5§	7.7 ± 0.8	9.0 ± 0.8§	8.8 ± 0.6
PVR, Wu	3.3 ± 0.3	2.9 ± 1.3	3.9 ± 0.5*	4.1 ± 0.4*	2.9 ± 0.4	5.6 ± 0.4*§#	3.0 ± 0.3
TPR, Wu	6.3 ± 0.6*	5.8 ± 0.6*	7.1 ± 1.1*	6.2 ± 0.5*	4.7 ± 0.4*	8.2 ± 0.6*#	3.3 ± 0.25
Cp, mL/mmHg	4.2 ± 0.28*	4.3 ± 0.3*	3.0 ± 0.7*#	2.9 ± 0.2*§	3.5 ± 0.2*	2.1 ± 0.1*§	6.2 ± 0.4
RC time, sec	0.8 ± 0.05*	0.67 ± 0.06*	0.98 ± 0.1*#	0.6 ± 0.02*§	0.55 ± 0.04*	0.69 ± 0.07*#	1.1 ± 0.03
EM, mm Hg	64 ± 5*	51 ± 5*	79 ± 9*#	105 ± 7*§	90 ± 7*§	124 ± 11*§#	21 ± 1.7
AWT, %	23 ± 1*	19 ± 1*	24 ± 1*	17 ± 1*§	18 ± 1*	15 ± 1 *§#	1.4 ± 1.3

Data are mean ± SEM

*Abbreviations*: *AWT* area wall thickness, *CI* cardiac index, *Cp* total pulmonary arterial capacitance, *EM* elastic modulus, *HR* heart rate, *mPAP and pPAP* mean and pulsatile pulmonary arterial pressure, respectively, *PAWP* pulmonary arterial wedge pressure, *PVR* pulmonary vascular resistance; SV: stroke volume, *RAP* right atrial pressure, *TPR* total pulmonary resistance

*p < 0.05 vs. Control; §*p* < 0.05 vs. COPD; #*p* < 0.05 vs. Non PH

**Fig. 1:**
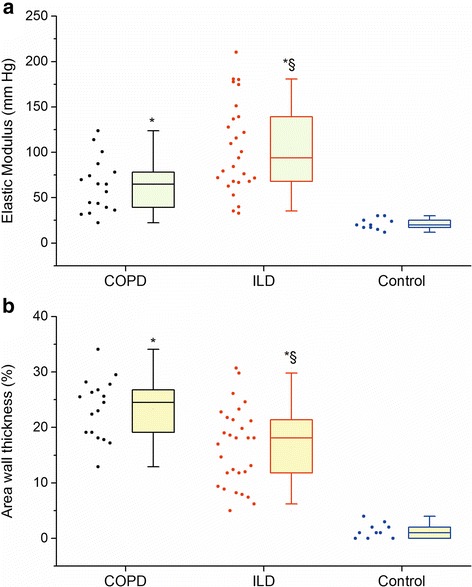
*Box plots* (median and percentiles 25/75) of Elastic Modulus **a** and area wall thickness **b** among the different groups. * *p* < 0.05 vs. Control. § *p* < 0.05 vs. COPD

Figure 1 showed the values of EM and AWT of COPD and ILD patients, which were significantly higher than controls, while ILD patients showed a significant higher EM and lower AWT than COPD patients (*p* < 0.05). All except one preLTx COPD patient presented higher EM (COPD: 22 to 124 mmHg; ILD: 33 to 210 mmHg) and AWT (COPD: 13 to 34; ILD: 5 to 31) than control subjects (EM: 12 to 30 mmHg and AWT: 1 to 4). Among the non-PH patients, 7/10 (COPD) and 10/15 (ILD) patients had a mPAP between 21–24 mmHg.

**Fig. 2:**
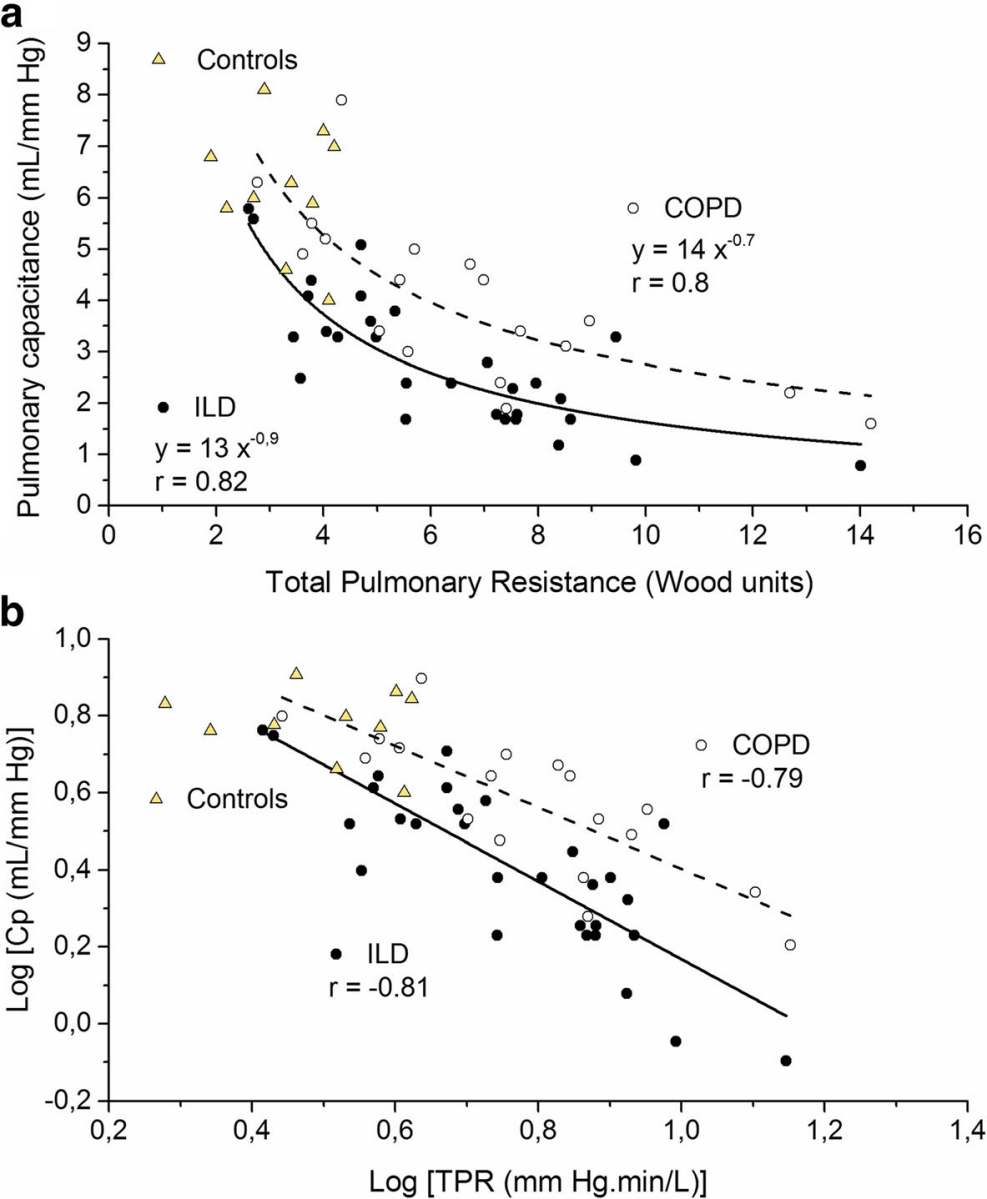
**a** Inverse relationship of Cp-TPR relationship and **b** Log TPR-log Cp plot. *Both curves* showed that ILD patients are shifted left downward

Figure 2 displays the hyperbolic relationship of TPR and Cp of both groups, showing that TPR-Cp curve is shifted downward left for ILD, with a reduced Cp value for a given TPR. The logarithmically transformed TPR-Cp plot showed that the slope of the linear regression for ILD and COPD were −1 and −0.8 respectively, indicating that TPR and Cp are tightly and inversely coupled in both groups of patients.

Mean diameter of the pulmonary arteries studied was similar among the different groups (COPD: 1.84 ± 1.3 mm; ILD: 2.03 ± 1.2 mm; controls: 1.91 ± 1.1 mm).

### Relationship between the severity of pulmonary hemodynamic and pulmonary arterial wall disease

Table 2 summarizes the hemodynamic and IVUS data for the patients with or without PH. Patients without PH of both groups showed a significant increased EM and AWT to control non-PH patients, which is associated with a higher mPAP and TPR and lower Cp and RC time (*p* < 0.05).

**Fig. 3:**
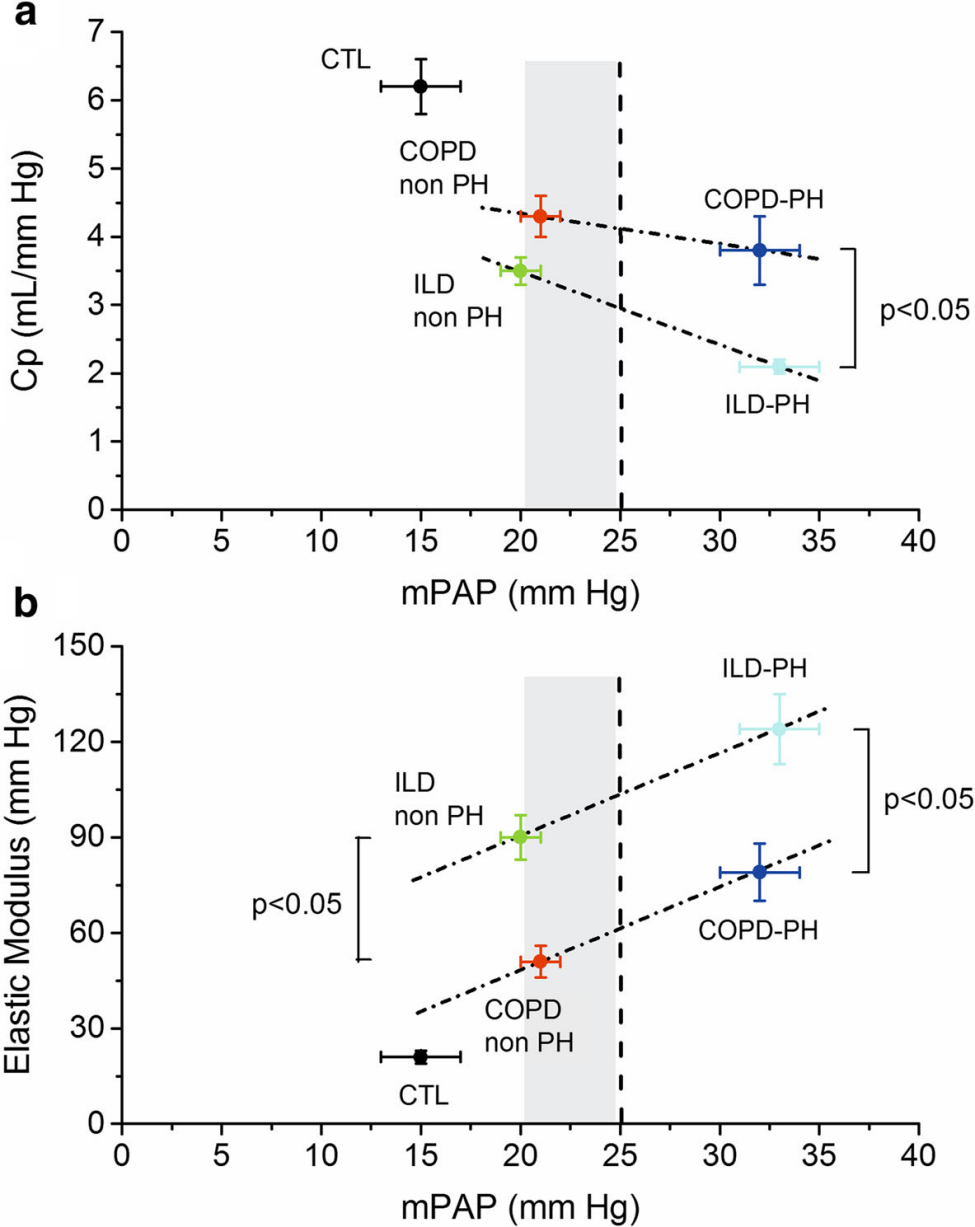
**a** Correlation between total pulmonary arterial capacitance (Cp) and mean pulmonary arterial pressure (mPAP) in COPD and ILD patients and **b** Correlation between elastic modulus (EM) and mPAP in COPD and ILD patients. The *dotted line* indicates the threshold for PH (mPAP = 25 mm Hg). The *gray rectangle* indicates mPAP between 21–24 mm Hg. *﻿CTL* Controls﻿

In COPD, both EM (*r* = 0.66, *p* < 0.01) and AWT (*r* = 0.6, *p* < 0.05) correlated with mPAP. By contrast, in ILD only EM (*r* = 0.63, *p* < 0.01) worsened with increasing mPAP. Both, Cp (*r* = −0.66, *p* < 0.01) and PVR (*r* = 0.43, *p* < 0.05) were correlated with EM in COPD. On the contrary, in ILD group, only PVR correlated with EM (*r* = 0.38, *p* < 0.05). Figure 3a and b show the dependence of the Cp and EM to the working mPAP in both groups. ILD patients showed lower Cp and higher EM than COPD, either in PH as non-PH patients.

### Relationship between lung function impairment and pulmonary hemodynamics and arterial wall disease in COPD and ILD

**Fig. 4:**
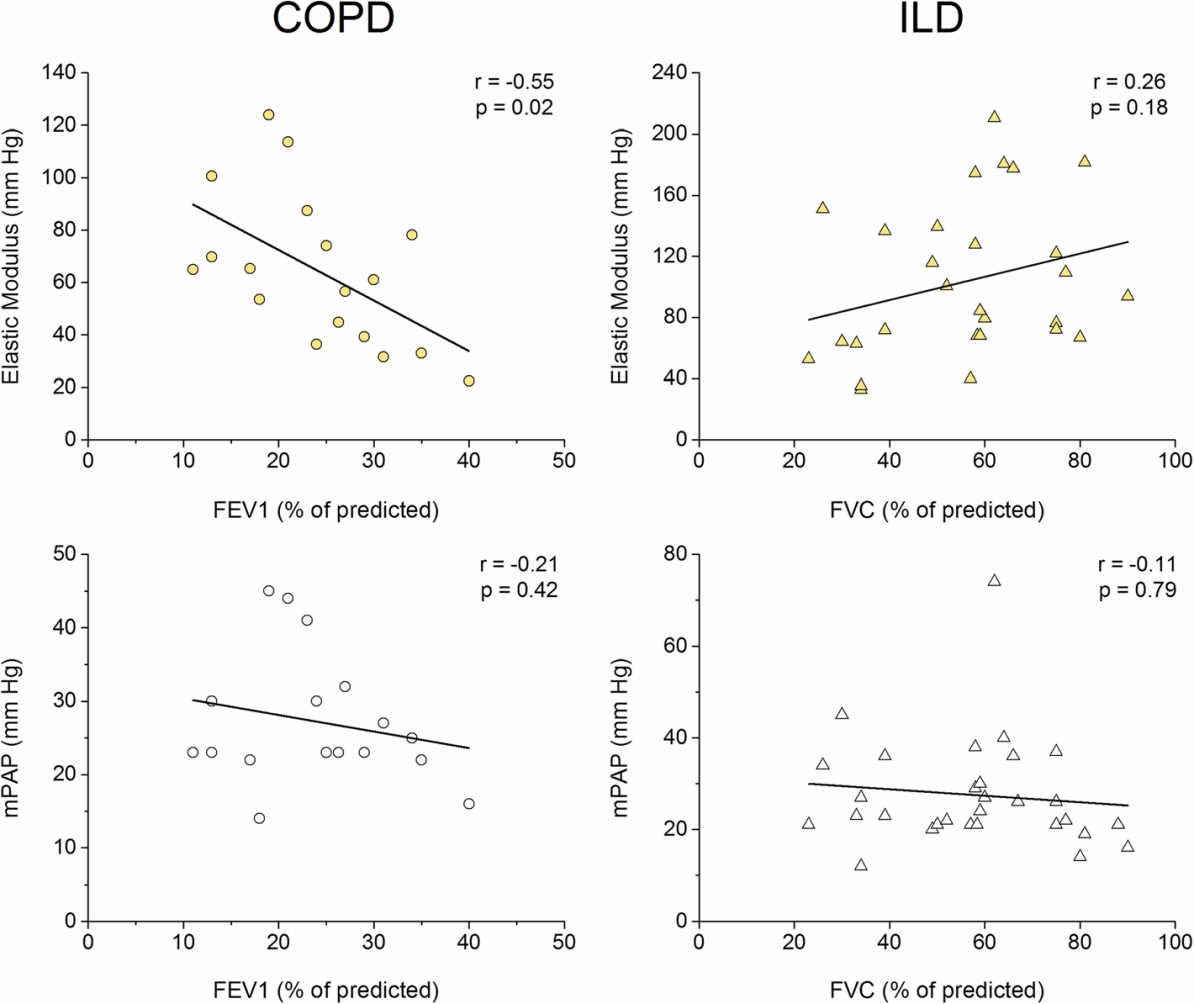
Correlations between lung function and Elastic Modulus and mean pulmonary arterial pressure (mPAP) of *COPD* (% of FEV1 predicted) and *ILD* (% of FVC predicted) patients

Both, COPD and ILD patients showed that PaO_2_ was correlated with mPAP (COPD: *r* = −0.44, *p* < 0.01; ILD: *r* = −0.35, *p* < 0.02) and EM (COPD: *r* = −0.35, *p* < 0.01; ILD: *r* = −0.43, *p* < 0.01). Only in COPD, partial pressure of carbon dioxide was correlated with mPAP (*r* = 0.37) and EM (*r* = 0.55) (*p* < 0.05). Figure 4 showed that only in COPD, EM and not mPAP, correlated to lung function impairment (FEV1).

## Discussion

We analyze the pulmonary vascular disease in candidates for LTx with advanced COPD and ILD. We have identified several issues having potential clinical relevance: (1) moderate to severe pulmonary vascular disease is present in almost all patients, even without PH and is related to hemodynamic severity; (2) patients with advanced COPD and ILD seem to have different pulmonary vascular disease; and (3) EM is related to the severity of airflow limitation in COPD patients.

### Pulmonary hemodynamics and pulmonary arterial wall disease in COPD and ILD patients

In the present cohort of advanced COPD and ILD preLTx patients, the prevalence of pre-capillary PH was 44% for COPD and 46% for ILD. This is in agreement with single center reports and the United Network for Organ Sharing database [6, 10, 25]. We found 27% of patients with PAWP > 15 mmHg which agrees with the Organ Procurement and Tissue Network (OPTN) database [4].

There are scarce data about the values of EM in healthy subjects. Sanz et al. estimated the main pulmonary artery (24–26 mm) stiffness by cardiac magnetic resonance imaging obtaining a median value of 32 mmHg [26]. Very recently, Shen et al. using IVUS imaging, assessed the EM of elastic pulmonary arteries of 4.4 ± 0.2 mm, obtaining a mean value of 44 ± 4 mmHg [27]. Both, age and range of mPAP of control patients are similar among the studies, excepting the control group of Shen’s work that included patients with connective tissue disease without PH, which can explain the higher values of EM secondary to some degree of subclinical vascular remodeling.

One of the main findings of the present study is the observation that patients without PH presented a significant pulmonary vascular disease with respect to controls. The increase of EM and AWT may indicate an early and significant pulmonary vascular disease in non-PH-lung disease patients. Therefore, the rise in resting mPAP above 25 mmHg is a late marker of the pulmonary vascular disease process [28]. Unlike several studies that attempt to detect some degree of pulmonary vascular disease through the exercise hemodynamic impairment [25, 29, 30], our results raise the assessment of PA wall behavior as an alternative tool to make the diagnosis of vasculopathy at a preclinical stage [31].

COPD patients showed a lower EM and higher Cp than ILD beyond mPAP; that was associated with a more preserved RC time and a right upward RC plot, such that Cp was higher for a given TPR. We analyzed the relationship between TPR and Cp and not PVR-Cp, to avoid the potential risk of misleading PAWP measurements [32, 33]. Probably the higher collagen fibers content of the arterial wall associated with the lung tissue fibrotic process could explain the greater stiffness with less thickness of the wall of ILD patients. This is in agreement with Hoffmann et al. [34] who have recently reported that pulmonary arterial remodeling in PH-COPD and PH-IPF patients is caused by different molecular mechanisms.

The higher RAP and PAWP of COPD than ILD patients could be related to a higher end-expiration intrathoracic pressure [35].

### Relationship between pulmonary arterial wall disease and lung function impairment in COPD and ILD

Multiple mechanisms have been postulated as underlying the development of PH in COPD but remain incompletely understood [36]. Both hypoxemia and hypercapnia showed a significant correlation with mPAP and EM in our pre-LTx COPD patients. Each one explains about 15 to 30% of mPAP and EM variation, reflecting the important role of chronic hypoxia and hypercapnia in vascular disease. The absence of correlation between the AWT and mPAP is in accordance to Kubo et al. [37], which reported that in contrast to mPAP at rest, mPAP during exercise was strongly related to pulmonary arterial wall thickness. Finally, the absence or weak correlation between PH and airway obstruction has been widely known and fits with our results [29, 38]. However, we obtained a strong relationship between FEV1 and EM (*r* = −0.55), highlighting that airflow limitation may be related to the severity of vasculopathy.

The pathogenesis of PH in ILD is also complex and multifactorial [39]. Correlations between PaO_2_ and mPAP and EM in the present study, illustrate the importance of hypoxia in vascular disease. The loss of pulmonary vasculature has been challenged as the main factor for the development of PH in ILD, especially in IPF [40]. Accordingly, previous and current studies did not show a positive relationship between the degree of restrictive physiology and PH [7, 10].

Regarding clinical differences, only ILD patients with PH have shown a 6MWT significantly lower than patients without PH, which may be related to the more severe wall remodeling.

### Study limitations

We measured intravascular pulmonary pressures at the end of expiration to minimize the effect of intrathoracic pressure changes during the respiratory cycle since the intra and extrathoracic pressures are equal (pleural pressure is closest to 0 = atmospheric pressure) and so, intravascular and transmural pressures are similar. Respiratory swings are usually more pronounced in advanced COPD patients, so, intrathoracic pressure is increased during expiration and may be greater than atmospheric pressure. Although averaging intravascular pulmonary pressures over the respiratory cycle may be better estimates of transmural pressures, particularly, during exercise in COPD patients [35], we employed end-expiratory measurements based on the recommendations for resting measurements [17]. We cannot discard an expiratory increase of RAP and PAWP that may explain the significantly higher values of the COPD patients [41]. Concomitantly, the pulse PAP and mPAP may decrease during spontaneous expiration in COPD patients at rest [42]. Therefore, we were careful to analyze the Cp (SV/pulse PAP) and EM (pulse PAP/IVUSp) for a given mPAP. This allowed to find out there are real differences in pulmonary vascular disease between COPD and ILD.

Although the non-PH control patients were not age- and sex-matched to COPD and ILD patients, age-related pulmonary arterial stiffness begins after ~ 50 years old [43].

We evaluate pre-LTx COPD and ILD patients, therefore, our findings cannot apply to all population of COPD and ILD. They also need external validity, since the present study shows the assessment of 46 patients from a single center.

Although the pathologic manifestations of each of the ILDs and their distribution within the lung parenchyma play a role in determining concomitant PH, more than half of PH-ILD patients included corresponding to IPF [8].

The absence of a significant correlation between the EM and the percentage of FVC predicted would allow discarding a prevail effect of the different perivascular pressures exerted by lung tissue connections on the elastic properties of the pulmonary arteries in ILD patients.

## Conclusions

The present study shows that PH is a late marker of the pulmonary vascular disease process in COPD and ILD candidates for LTx. PA stiffness (assesses by the EM) and the relative area of wall thickness can make the diagnosis of pulmonary vasculopathy at a preclinical stage. Patients with advanced COPD and ILD appear to have a different pulmonary vascular disease. ILD patients showed a worse pulmonary vascular disease with higher EM and lower Cp for a given mPAP. Airflow limitations in COPD but not restriction impairment in ILD patients would be associated with the pulmonary vascular disease. Further research is needed to identify optimal screening methods for pulmonary vascular disease in this patient population and to explore specific therapeutic options.

## Abbreviations

6MWT: Six-minute walking test
AWT: Area of wall thickness
CO: Cardiac output
COPD: Chronic obstructive pulmonary disease
Cp: Total pulmonary arterial capacitance
DLCO: Diffusion capacity for carbon monoxide
EM: Elastic modulus
FEV1: Forced expiratory volume in one second
FVC: Forced vital capacity
ILD: Interstitial lung disease
IVUSp: Intravascular ultrasound pulsatility
LTx: Lung transplantation
mPAP: Mean pulmonary arterial pressure
PAS: Pulmonary arterial stiffness
PAWP: Pulmonary arterial wedge pressure
PH: Pulmonary hypertension
PVR: Pulmonary vascular resistance
RAP: Right atrial pressure
TPR: Total pulmonary resistance
